# Effects of Naringin on Proliferation and Osteogenic Differentiation of Human Periodontal Ligament Stem Cells In Vitro and In Vivo

**DOI:** 10.1155/2015/758706

**Published:** 2015-05-20

**Authors:** Lihua Yin, Wenxiao Cheng, Zishun Qin, Hongdou Yu, Zhanhai Yu, Mei Zhong, Kemo Sun, Wei Zhang

**Affiliations:** ^1^School of Life Sciences, Lanzhou University, Lanzhou, Gansu, China; ^2^Department of Oral Implantology, School/Hospital of Stomatology, Lanzhou University, No. 199 Donggang West Road, Chengguan, Lanzhou, Gansu, China; ^3^School/Hospital of Stomatology, Lanzhou University, No. 199 Donggang West Road, Chengguan, Lanzhou, Gansu, China; ^4^Shanghai Institute for Advanced Immunochemical Studies (SIAIS), ShanghaiTech University, No. 99 Haike Road, Pudong, Shanghai 201210, China

## Abstract

This study is to explore the osteogenesis potential of the human periodontal ligament stem cells (hPDLSCs) induced by naringin in vitro and in vitro. The results confirmed that 1 *μ*M naringin performs the best effect and a collection of bone-related genes (*RUNX2*, *COL1A2*, OPN, and OCN) had significantly higher expression levels compared to the control group. Furthermore, a typical trabecular structure was observed in vivo, surrounded by a large amount of osteoblasts. These results demonstrated that naringin, at a concentration of 1 *μ*M, can efficiently promote the proliferation and differentiation of hPDLSCs both in vitro and in vivo.

## 1. Introduction

Periodontal disease is a chronic infectious disease that occurs in the periodontal tissue (gums, periodontal ligament, cementum, and alveolar bone), with the main clinical manifestations of alveolar bone and cementum absorption caused by tooth loss. Despite some reports of successful bone healing with autografts and allografts, which are the most common clinical treatments for large nonunion defects, these techniques have not yet been able to meet all clinical needs [1]. In 2004, Seo et al. [2] reported in The Lancet that adult stem cells from the human periodontal ligament induce conditions in mineralization through its osteogenic differentiation capacity, by inducing conditions of mineralization that have osteogenic-differentiation capacity. The cells are able to form cementum, a periodontal ligament-like structure in the dorsal skin of immunodeficient mice; these structures were later named human periodontal ligament stem cells (hPDLSCs) [2]. hPDLSCs are a class of perivascular accumulations with characteristics of stem cells, which can theoretically differentiate into fibroblasts, osteoblasts, cementum cells, and osteoclasts and break cementum cells [3–5].

**Figure 1:**
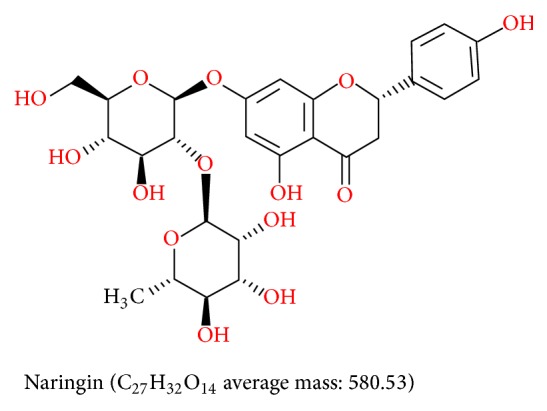
The chemical structure of naringin.

Naringin is a hydrochlorothiazide flavonoid, the main active ingredient of Chinese medicine extracts of Drynaria, and has a known chemical structure (Figure 1). The pharmacological roles of naringin in promoting human mesenchymal stem cell proliferation and differentiation [6–8] are well documented. However, there are few studies on therapeutic evaluation of naringin on human periodontal ligament stem cells. Nanohydroxyapatite (nHAC) scaffold is a nanosized bionic bone material and very close to natural bone in its nanostructure. It has a high porosity of approximately 80–90% and a pore size of approximately 100–600 *μ*m, which can be degraded by more than 80% in 6 months. Therefore, it has the desired porosity, biocompatibility, and degradation rate [9–11]. Most importantly, we have demonstrated that naringin-stimulated hPDLSCs can proliferate on nanohydroxyapatite scaffolds and develop into mineralized tissues when grafted into mice.

In the present study, we explored the effect of naringin on proliferation and osteogenic differentiation of human periodontal ligament stem cells. After determining the optimal naringin concentration for osteogenic differentiation, we put hPDLSCs composite of nanohydroxyapatite scaffold in immunodeficient mice to confirm the osteogenic effect in vivo, providing a theoretical basis for further research investigation and for clinical practice.

## 2. Materials and Methods

### 2.1. The Chemical Structure of Naringin

Naringin (Figure 1) is a dihydrotestosterone flavonoid. Because there is no conjugation between the A and B rings, naringin displays a variety of biological activities and pharmacological effects.

### 2.2. Isolation and Culture of Human Periodontal Ligament Stem Cells

Human periodontal ligament stem cells were isolated and cultured according to a previously reported protocol with minor modifications [2]. In brief, human premolars (*n* = 20) were extracted for orthodontic reasons from 10 healthy adult volunteers. Periodontal ligament tissues were separated from the root surface using a scalpel and were minced into the smallest size possible. The minced periodontal ligament tissues were digested for 45 min in digestive enzymes (3 mg/mL collagenase type I [Sigma, USA] and 4 mg/mL Dispase II [Sigma, USA]) at 37°C. Single-cell suspensions were seeded onto cell culture dishes (BD, USA) containing growth medium of a-MEM (Hyclone, USA) supplemented with 20% fetal bovine serum (FBS; Gibco, USA) and then incubated at 37°C with 5% CO_2_. Single-cell colonies were observed and passage 0 (P0) cells were cultured. Then, passages 3 through 5 (P3–P5) cells were used for this study.

### 2.3. Immunohistochemistry

hPDLSCs were seeded into 12-well plates (5 × 10^3^ cells/well) for 24 h. The cells were fixed in 4% paraformaldehyde for 15 min and blocked at room temperature for 30 min with 1% BSA. Then, the cells were incubated overnight at 4°C with rat-anti-Stro-1 and CD146 (Abcam, USA). After washing with PBS, sheep anti-Rat IgG secondary antibody was then added and incubated with the fixed hPDLSCs at 37°C for 1 h. The results were detected by fluorescence microscope (ECLIPSE Ti-E, Nikon, Japan), with no secondary antibody used as a blank control.

### 2.4. Colony-Forming Unit Fibroblastic and Differentiation Assay

To assess colony-forming efficiency, P3 hPDLSCs were fixed with 4% formalin and then stained with 0.1% toluidine blue. Aggregates of 50 or more cells were scored as a colony. Calcium accumulation was induced on day 21 and then detected by staining with 2% Alizarin Red S. The medium was refreshed twice weekly. After 4 weeks, cells were fixed with 4% paraformaldehyde and stained with Oil Red-O for detection of lipid droplets.

### 2.5. MTT Proliferation Assay

Stock dimethyl sulfoxide (DMSO, Sigma, USA) culture fluid (Hyclone, China) was prepared by dissolving 20 mg naringin (National Institute for the Control of Pharmaceutical and Biological Products, China) in DMSO (345 *μ*L). The stock solution was then 10-fold serially diluted (10 nM to 100 *μ*M) into *α*-MEM plus 10% fetal bovine serum [12]; 2 × 10^3^ hPDLSCs/well were seeded in 96-well plates in *α*-MEM containing 10% FBS. Then, the media were refreshed with *α*-MEM containing 10% FBS and various concentrations of naringin after 24 h. hPDLSCs were induced with icariin for 1 to 5 days; 20 *μ*L of MTT (tetrazolium salt) solution (5 mg/mL in PBS, pH = 7.4) was added to each well after medium removal. After incubation for 4 h, the supernatant was discarded and 150 *μ*L of DMSO was added into each well to fully dissolve crystals through 10 min of oscillation. The absorbance at 490 nm was determined by an enzyme-linked immunosorbent assay reader (Elx 800, Bio-Tek, Winooski, VT, USA). Cells in *α*-MEM containing 10% FBS without naringin were used as a blank control. Cell viability was determined by comparing the OD value of the treatment samples with the blank control.

### 2.6. Measurement of Alkaline Phosphatase Activity

The cells were seeded in 96-well plates (2 × 10^3^ cells/well) and cultured for 3, 5, and 7 days (37°C, 5% CO_2_). The cells were lysed by 0.1% Triton X-100 (Ambion by Life Technologies). The ALP activity in the lysates was determined by p-nitrophenyl phosphate (PNPP) hydrolysis method using a commercial assay kit (Nanjing Jiancheng Bioengineering Institute, Nanjing, China). Absorbance at 520 nm was measured and data were normalized for total protein content by Bradford law, which was measured the amount of alkaline phosphatase in cells.

### 2.7. Osteogenic Differentiation of hPDLSCs Induced by Naringin

hPDLSCs cells were seeded in 96-well plates at a density of 2 × 10^4^ cells/well in *α*-MEM containing 10% FBS without osteogenic supplements. After 24 h, naringin was added at concentrations ranging from 10 nM to 1 *μ*M. Cells cultured without naringin were used as a blank control. At day 21, the cells were washed twice with PBS, fixed in 95% ethanol for 10 min, and stained by 0.1% Alizarin Red S (pH 8.3) at 37°C for 30 min. After washing with distilled water, calcium nodules were observed under an optical microscope.

### 2.8. RNA Isolation, cDNA Synthesis, and Quantitative Real-Time PCR Analysis

After 7 days' culture, the total RNA was extracted using Trizol RNA extraction reagent (TAKARA, Dalian, Liaoning, China). cDNA was synthesized with PrimeScript RT Master Mix (TAKARA). RT-PCR was performed by ABI 7300 Real-Time PCR System using Premix Ex Tag (TAKARA). The cycling parameters used were as follows: denaturation at 95°C for 15 min and 30 amplification cycles (15 s at 95°C, 25 s at 60°C, and 10 s at 72°C). The primers were* RUNX2*,* COL1A2*, osteopontin (OPN), and osteocalcin (OCN) (Table 1). Expression of *β*-actin was used as an internal control.

### 2.9. In Vivo Transplantation

The protocol of care, maintenance, and treatment of animals in the present study was approved by the Institutional Animal Care Committee of Lanzhou University. Before transplantation, a total of 4.0 × 10^6^ hPDLSCs were inoculated onto the medium and were refreshed with naringin fluid culture (1 *μ*M), and the cells were incubated for a week. The 6-week-old immunocompromised Beige mice (Vitalriver, Beijing, China) were randomly divided into 2 groups: mice transplanted with drug-treated cells and mice without drug-treated cells. The amount of local anesthetic was calculated according to the mice body weight. When the transplantation began, the operation area was disinfected with povidone-iodine and the 3% chloral hydrate (1 mL/100 g) was injected intraperitoneally for the full anesthesia. After that, we created a full-thickness incision in the mouse back skin and transplanted the induced cells into it. Surgical sutures were removed a week later.

### 2.10. Immunohistochemical Analysis of Transplanted Tissues

The transplants were harvested after 8 weeks of transplantation and fixed with 4% paraformaldehyde for 24 hours. Hematoxylin and Eosin (H&E) staining and immunohistochemical staining were performed on tissue sections. Primary antibodies used for IHC staining were rabbit anti-human bone gamma-carboxyglutamate protein (BGP) antibody (1 : 200 dilution, Boster, Wuhan, China) and rabbit anti-human osteopontin (OPN) antibody (1 : 200 dilution, Boster, Wuhan, China). After washing, the samples were treated with FITC-labeled goat anti-rabbit IgG (1 : 64 dilution, Boster, Wuhan, China). Images of the newly mineralized structures were obtained from fluorescence microscope (ECLIPSE Ti-E, Nikon, Japan).

### 2.11. Statistical Analysis

All measurements were collected with *N* = 6 and expressed as means ± standard deviations. SPSS 18.0 was used for data analysis using analysis of variance (ANOVA) followed by *t*-test, with statistically significant values defined by *P* values <0.05.

## 3. Results and Discussion

### 3.1. Isolation and Culture of Human Periodontal Ligament Stem Cells (hPDLSCs)

**Figure 2:**
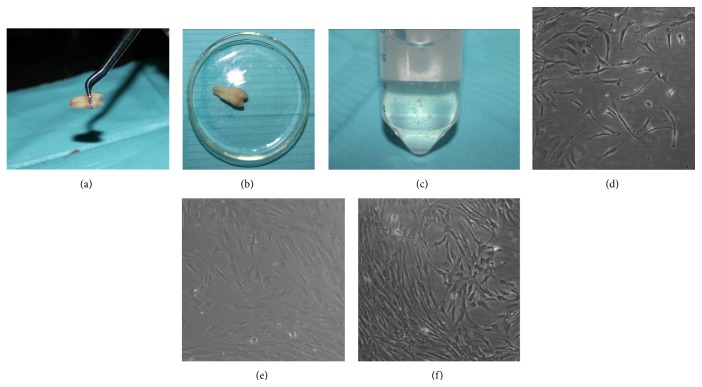
Primary culture of hPDLSCs ((a), (b), and (c)). (d) 7 d (×100), (e) 10 d (×100), and (f) 14 d (×40).

Periodontal ligament tissues were separated from the root surface using a scalpel and were minced into the smallest pieces (Figures 2(a), 2(b), and 2(c)). After 7 days of culture, individual cells appeared (Figure 2(d)). On day 10, we observed that the adherent cells showed a fusiform shape (Figure 2(e)). On day 14, hPDLSCs proliferated actively and exhibited directionality of the cell arrangements (Figure 2(f)).

### 3.2. Characterization of Isolated hPDLSCs

**Figure 3:**
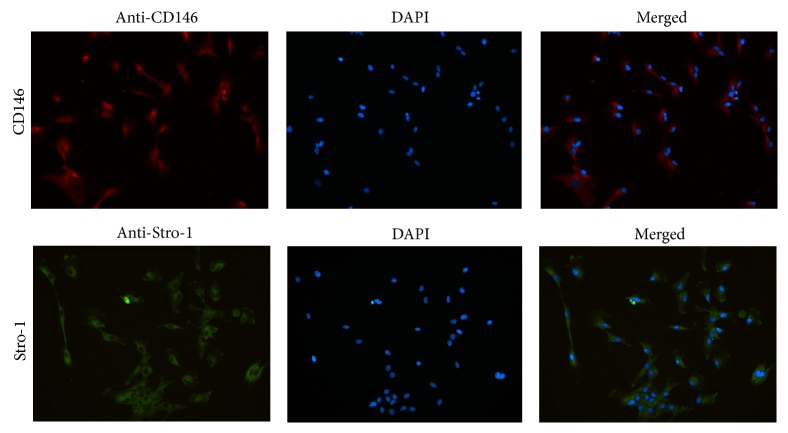
Immunofluorescence staining of CD146 and Stro-1 on hPDLSCs.

**Figure 4:**
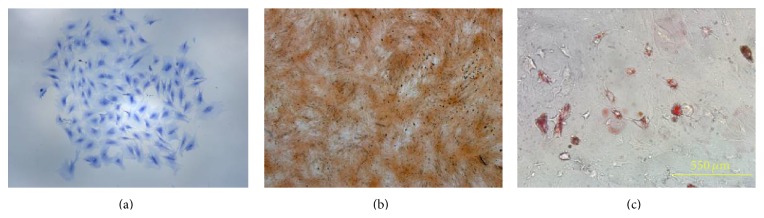
(a) A single colony stained with 0.1% toluidine blue (×100). (b) Mineralization assay: Alizarin Red S (×100). (c) Differentiation of PDLSCs into adipocytes (×100).

Stro-1 and CD146 are typical surface markers of the periodontal ligament stem cells. Cell surface marker (Stro-1) is green fluorescent by FITC (Figure 3). Cell (CD146) is green fluorescent by FITC. Toluidine blue-positive staining of the hPDLSCs indicated the presence of glycosaminoglycans in cartilage matrix (Figure 4(a)). During the course of osteogenic differentiation, mineralized nodules were formed, as revealed using Alizarin Red Staining on day 21 (Figure 4(b)). With regard to adipogenic differentiation, cells showed lipid droplets after 21 days of adipogenic differentiation (Figure 4(c)).

### 3.3. Cell Proliferation Assay

**Figure 5:**
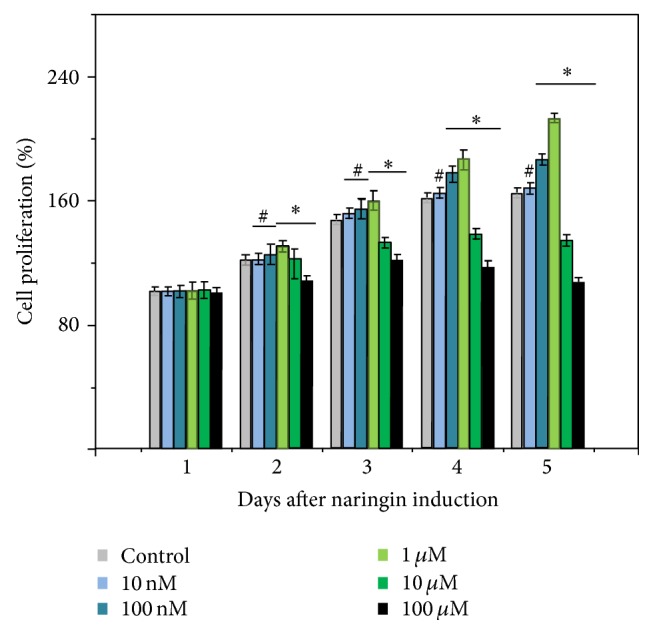
Proliferation of hPDLSCs stimulated by naringin. Data were shown as the mean ± SD (*n* = 6). *∗* is compared with control group, *P* < 0.05. # indicates no significant difference. There is no significant difference between the blank control and the treatment group at the concentration of 10 nM naringin. At day 4, with an over 20% increase in proliferation rate, the difference between the blank control and the treatment group was significant (*P* < 0.05) when naringin concentration was increased to 100 nM. The treatment group consistently exhibited a significant increase in proliferation rate over the blank control (*P* < 0.05) at the concentration of 1 *μ*M naringin. However, when we further increased naringin concentration up to 100 *μ*M, we found a detrimental effect.

During the course of cell proliferation study of hPDLSCs, a general trend in cell proliferation was observed. Moreover, a dose-dependent behavior of naringin concentration on cell proliferation was exhibited (Figure 5). At the concentration of 10 nM naringin, we did not see a significant difference between the blank control and the treatment group. When naringin concentration was increased to 100 nM after day 4, the difference between the blank control and the treatment group was significant (*P* < 0.05), with an over 20% increase in proliferation rate for the cells treated with naringin. At the concentration of 1 *μ*M naringin, the treatment group consistently exhibited a significant increase in proliferation rate over the blank control (*P* < 0.05). However, when we further increased naringin concentration up to 100 *μ*M, we found that naringin at these concentrations had a detrimental effect on cell proliferation. Therefore, we only investigated the therapeutic effect of naringin at the concentration ranging from 10 nM to 1 *μ*M in the following experiment.

### 3.4. Measurement of Alkaline Phosphatase Activity

**Figure 6:**
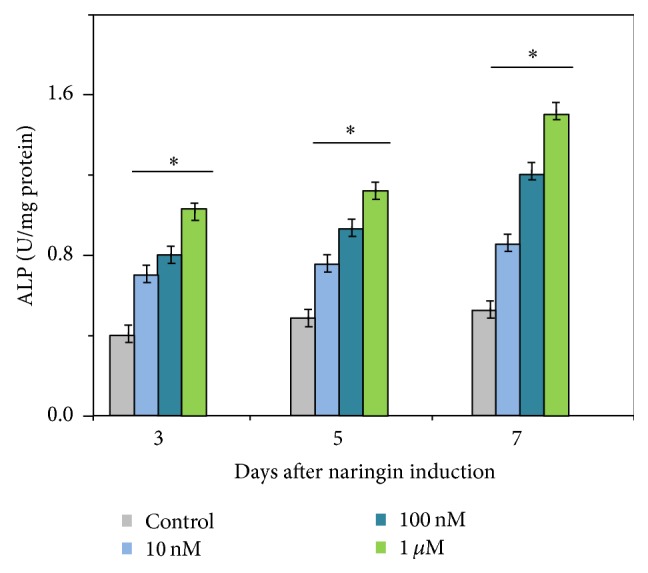
The expression of ALP Data was shown as the mean ± SD (*n* = 6). *∗* is compared with control group, *P* < 0.05. With increasing concentrations of naringin from 10 nM to 1 *μ*M, a substantial elevation in ALP expression was observed from days 3 to 7 in the treatment group compared to the blank control (*P* < 0.05). Furthermore, the ALP expression was positively correlated with the concentration of naringin, but naringin of 10 nM showed limited effects. Among them, 1 *μ*M naringin exhibited the most significant effect at day 7.

Alkaline phosphatase activity is a well-defined measure of osteogenesis and can reflect the degree of differentiation. With increasing concentrations of naringin from 10 nM to 1 *μ*M, a substantial elevation in ALP expression was observed from days 3 to 7 and compared to the blank control (*P* < 0.05). Moreover, the ALP expression level was positively correlated with the concentration of naringin, but naringin at 10 nM showed a little effect on the expression of ALP from days 3 to 7. On the 7th day, 1 *μ*M naringin showed the most significant differences on alkaline phosphatase activity (Figure 6).

### 3.5. Osteogenic Differentiation

**Figure 7:**
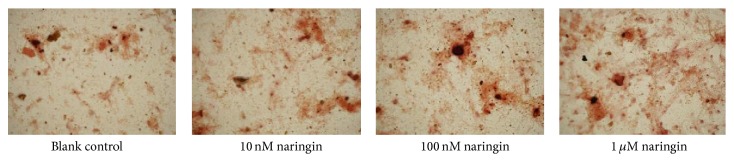
The image of mineralized deposits.

PDLSCs were cultured in osteogenic differentiation medium for 14 days, and hPDLSCs osteogenic differentiation status was determined by staining with Alizarin Red S. With the treatment of naringin (10 nM to 1 *μ*M), formation of calcium nodules was found to be increased, especially significant with the treatment of naringin at the concentration of 1 *μ*M (Figure 7).

### 3.6. RT-PCR Analysis of Bone-Related Genes

**Figure 8:**
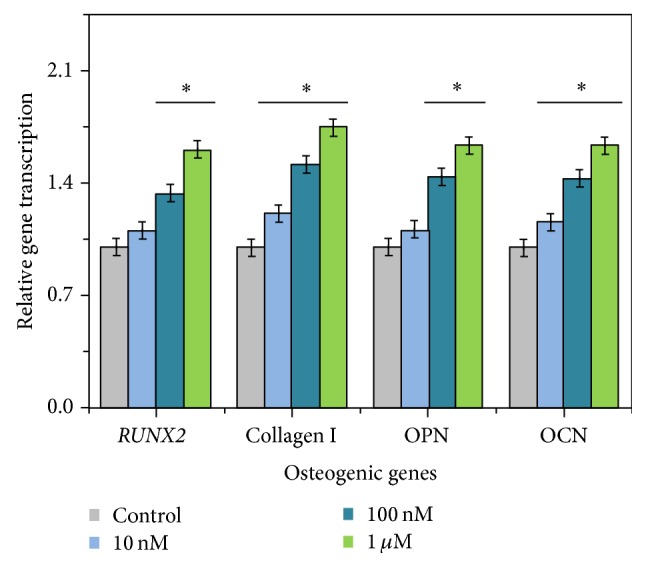
RT-PCR for the determination of osteoblastic marker expression was performed on day 14 after induction. Data were presented as the mean ± SD (*n* = 6). *∗* is compared with control group, *P* < 0.05.* RUNX2*, collagen I, OPN, and OCN expression were significantly determined in the treatment groups and were positively correlated with the naringin concentration. Among them, 1 *μ*M naringin showed the most positive effect.

Gene expression of standard osteogenic differentiation markers (such as* RUNX2*, collagen I, OPN, and OCN) was found to be positively correlated with the naringin concentration, with the highest gene expression over 1.5-fold by the treatment of 1 *μ*M naringin (Figure 8).

### 3.7. In Vivo Implantation Results

**Figure 9:**
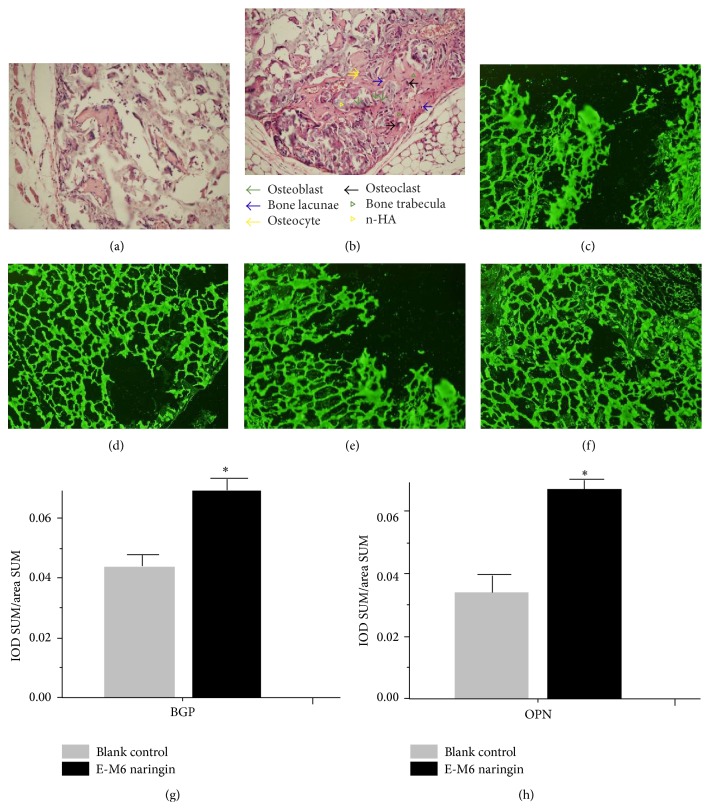
(a) Newly formed bone and dense connective tissue around the nanohydroxyapatite in the control group (×100), (b) in naringin 1 *μ*M group, newly formed bone and dense connective tissue around the nanohydroxyapatite (×100), (c) FITC fluorescence expression of BGP in the control group (×100), (d) in naringin 1 *μ*M group, FITC fluorescence expression of BGP (×100), (e) FITC fluorescence expression of OPN in the control group (×100), (f) in naringin 1 *μ*M group, FITC fluorescence expression of OPN (×100), (g) average optical density IOD SUM/area SUM of BGP, and (h) average optical density IOD SUM/area SUM of OPN. Data were presented as the mean ± SD (*N* = 6). *∗* is compared with control group, *P* < 0.05.

H&E staining suggested the generation of new bone tissue (Figures 9(a) and 9(b)) for both the control group and the treatment group. However, the differences between the 2 groups were significant (Figure 9(b)). Early osteoblast development and trabecular bone tissue surrounding the nanohydroxyapatite could only been seen in the treatment group. Immunofluorescence staining suggested the expression and excretion of BGP and OPN by osteogenic differentiation of hPDLSCs surrounding the nanohydroxyapatite. Image-Pro Plus 6.0 was then used for quantitative analysis of fluorescence intensity, and average optical density (IOD SUM/area SUM) was used as a measure of expression. The treatment group exhibited an over 50% increase in expression for both BGP and OPN.

### 3.8. Discussion

Periodontal disease, with chronic inflammation-induced collapse of periodontal ligament tissue, frequently can result in alveolar bone damage and tooth loss in adults. Previous studies demonstrated that MSCs-based periodontal therapy can inhibit the immune response and promote bone regeneration [13, 14]. PDLSCs are isolated from periodontal ligament. They have multiple differentiation potential, especially the cementum/periodontal ligament-like structure. So, they are often considered as the first candidate for periodontal tissue restoration [15].

Naringin, a compound with a well-defined chemical structure, is one of the main active ingredients of Drynaria, a traditional Chinese medicine that is widely used for promoting efficient generation of bone tissue [16]. Naringin is one of the flavonoid components that are considered natural antioxidants. Kanno et al. examined the effects of naringin on H_2_O_2_-induced cytotoxicity and apoptosis in mouse leukemia P388 cells. Their results indicate that naringin is a useful drug with antioxidant and antiapoptotic properties [17]. Naringin also improved antioxidant status by increasing the activity of antioxidant enzymes and nonenzymatic antioxidant levels, using its antioxidant and antiapoptotic role to protect nerve cells [18]. In another study on the mechanisms underlying cardioprotective effects of naringin, the researchers found that treatment with naringin can significantly inhibit HG-induced apoptosis by attenuating mitochondrial dysfunction and modulating the activation of the p38 signaling pathway [19].

A good proliferation potential is prerequisite for cell differentiation [12, 20]. The results of the present study showed that 1 *μ*M amount of naringin had the best performance. Based on cellular proliferation assays, we found that, with the increase in drug concentration, the cells showed a better growth trend. However, interestingly, when the drug concentration continued to increase to 10 *μ*M, there was a negative impact on cell growth. We deduced that the 10 *μ*M of naringin would inhibit the cell growth. Other studies showed the similar results. Dai and colleagues [6] examined the stimulative effects of naringin (0–200 ng/mL) on the proliferation of hBMSCs; their results showed that a certain concentration (1–100 *μ*g/mL) of the naringin may enhance the proliferation and osteogenic differentiation of hBMSCs. However, the number of cells in the group of 200 *μ*g/mL naringin decreased rapidly, indicating that the naringin of this (or higher) concentration was seriously poisonous to cell growth [6]. Liu et al. induced osteogenic differentiation of hAFSCs by naringin for their therapeutic potential [21]; they found that naringin can increase the proliferation and alkaline phosphatase activity (ALP) of hAFSCs in the range of 1–100 *μ*g/mL, while an inhibitory effect was observed at 200 *μ*g/mL. Thus, Liu and colleagues confirmed that naringin had an identified effect on the osteogenic differentiation of hAFSCs [21].

*RUNX2* plays an important role in osteoblast differentiation and bone formation and has been identified as a target of mechanical stress-induced signaling in osteoblastic cells. By the activation of MAPK signal transduction pathways and Ras/Raf-dependent ERK1/2 activation,* RUNX2* and osteoblasts can be activated [22, 23]. Furthermore, the differentiation stages of mature osteoblasts can synthesize and secrete OPN and OCN, reflecting the expression levels of the osteoblast [24]. In the present study, significant expressions of* RUNX2*, OPN, OCN, and collagen I were detected indicating the active differentiation of hPDLSCs.

The results of proliferation and osteogenic differentiations in vivo are consistent with the process in vitro. Apparent trabecular bone, which was surrounded by amount of osteoblasts on the surface, was observed to embed into nanohydroxyapatite. Meanwhile, the H&E staining showed that the maturity of trabecula in the experimental group was better than in the control group. Furthermore, the results of immunofluorescence demonstrated that the average optical density of OPN and OCN in experimental group was greater compared to the control group. Taken together with the intervention of naringin, osteogenic differentiation of hPDLSCs was accelerated and the formation of hard tissue was benefited within a short period of time [25].

## 4. Conclusion

In this study, naringin was used to stimulate the hPDLSCs in vitro and in vivo. The results demonstrated that naringin, as the main active compound of Rhizoma Drynariae, has an identified effect on the proliferation and osteogenic differentiation of hPDLSCs. The current in vivo study is of 2-month duration; therefore, a long-term evaluation of therapeutic effects of naringin needs to be conducted in the future.

## Figures and Tables

**Table 1:** RT-PCR primers (human).

Gene	GenBank accession	Primer
*RUNX2 *	Hs00231692	F5′-CACTGGCGCTGCAACAAGA-3′
		R5′-CATTCCGGAGCTCAGCAGAATAA-3′

*COL1A2 *	Hs00164004	F5′-GAGGGCAACAGCAGGTTCACTTA-3′
		R5′-TGGGCCAATGTCCACAAAGA-3′

OPN	NM001040060	F5′-GATGAATCTGATGAACTGGTCACT-3′
		R5′-GGTGATGTCCTCGTCTGTAGCA-3′

OCN	NM199173	F5′-GACGAGTTGGCTGACCACA-3′
		R5′-CAAGGGGAAGAGGAAAGAAGG-3′
